# Repetitive DNAs and Karyotype Evolution in Phyllostomid Bats (Chiroptera: Phyllostomidae)

**DOI:** 10.3390/biom15091248

**Published:** 2025-08-29

**Authors:** Geize Aparecida Deon, Tariq Ezaz, José Henrique Forte Stornioli, Rodrigo Zeni dos Santos, Anderson José Baia Gomes, Príncia Grejo Setti, Edivaldo Herculano Correa de Oliveira, Fábio Porto-Foresti, Ricardo Utsunomia, Thomas Liehr, Marcelo de Bello Cioffi

**Affiliations:** 1 Departamento de Genética e Evolução, Universidade Federal de São Carlos, São Carlos 13565-905, São Paulo, Brazil; geizeadeon@gmail.com (G.A.D.); princiasetti@gmail.com (P.G.S.); mbcioffi@ufscar.br (M.d.B.C.); 2 Institute of Applied Ecology, Faculty of Science and Technology, University of Canberra, Australian Capital Territory, Canberra 2617, Australia; tariq.ezaz@canberra.edu.au; 3 Faculdade de Ciências, Universidade Estadual Paulista, Bauru 17033-360, São Paulo, Brazil; jose.henrique@unesp.br (J.H.F.S.); rodrigo.zeni@unesp.br (R.Z.d.S.); fp.foresti@unesp.br (F.P.-F.); ricardo.utsunomia@unesp.br (R.U.); 4 Department of Genetics, Institute of Biological Sciences and Health, Rural Federal University of Rio de Janeiro, Seropédica 23897-000, Rio de Janeiro, Brazil; 5 Instituto Federal de Educação, Ciência e Tecnologia, Abaetetuba 68440-000, Pará, Brazil; anderson.gomes@ifpa.edu.br; 6 Laboratório de Citogenômica e Mutagênese Ambiental, Instituto Evandro Chagas, Ananindeua 67030-000, Pará, Brazil; ehco@ufpa.br; 7 Instituto de Ciências Exatas e Naturais, Universidade Federal do Pará, Belém 66075-110, Pará, Brazil; 8 Institute of Human Genetics, Jena University Hospital, Friedrich Schiller University Jena, 07747 Jena, Germany

**Keywords:** cytogenomics, satellitome, telomeric repeats, multiple sex chromosomes

## Abstract

Bats are great models for studying repetitive DNAs due to their compact genomes and extensive chromosomal rearrangements. Here, we investigated the repetitive DNA content of two phyllostomid bat species, *Artibeus lituratus* (2*nn* = 30♀/31♂) and *Carollia perspicillata* (2*n* = 20♀/21♂), both harboring a multiple XY_1_Y_2_ sex chromosome system. Satellite DNA (satDNA) libraries were isolated and characterized, revealing four and ten satDNA families in *A. lituratus* and *C. perspicillata*, respectively. These sequences, along with selected microsatellites, were in situ mapped onto chromosomes in both species and phylogenetically related taxa. SatDNAs showed strong accumulation in centromeric and subtelomeric regions, especially pericentromeric areas. Cross-species mapping with *C. perspicillata*-derived probes indicated terminal localization patterns in other bat species, suggesting conserved distribution. Microsatellites co-localized with 45S rDNA clusters on the neo-sex chromosomes. Additionally, genomic hybridization revealed a male-specific signal on the Y_1_ chromosome, pointing to potential sex-linked repetitive regions. These findings confirm that bat genomes display relatively low amounts of repetitive DNA compared to other mammals and underscore the role of these elements in genome organization and sex chromosome evolution in phyllostomid bats.

## 1. Introduction

Repetitive DNA elements can be broadly classified into two major categories based on their distribution: dispersed elements, such as transposable elements (TEs), and tandem repeats, which include multigene families as well as three subclasses defined by the length of their repeat units—satellite, minisatellite, and microsatellite sequences [1,2]. Except for multigene families, tandem repeats are usually non-coding sequences organized in long arrays that evolve at high rates, being considered the fastest mutating repetitive DNA class [3]. Typically, these sequences accumulate in pericentromeric and centromeric heterochromatin but can also be present in euchromatic regions [4,5,6,7], and, in certain instances, linked to chromosomal rearrangement processes [8,9,10]. Furthermore, the proportion of repetitive DNA sequences can vary significantly across the genomes of vertebrates, particularly in mammals, resulting in variations in genome size among different species [11].

Encompassing roughly 1474 living species across 21 families and 236 genera, Chiroptera represents the second most-speciose mammalian order, surpassed only by Rodentia [12]. With estimated genome sizes spanning from 1.6 to 3.54 Gb, bats displays the smallest reported genomes among mammals [11]. Their small genome sizes are frequently linked to physiological adaptations, such as flight [13,14], as the genomes of flying mammals and birds are regarded as more compact than those of their flightless counterparts [15]. One proposed explanation for this reduction is a decreased on their repetitive DNA content [16,17,18,19]. However, research on repetitive DNAs in bat genomes is scarce and has primarily focused on in situ mapping of ribosomal DNAs (rDNA), telomeric repeats, and certain transposable elements (as reviewed in [20]).

Among bat families, Phyllostomidae Gray, 1825, which includes the New World leaf-nosed bats, is the third-largest family, comprising approximately 200 species distributed across 60 genera, all endemic to the Americas [21]. This group is known for its diversity in ecological niches, morphological variation, and extensive chromosomal diversification [22,23]. Therefore, diploid numbers (2*n*) of this family widely vary, ranging from 14 to 46 chromosomes, with the well-established simple XY system coexisting with multiple sex chromosome systems, including species of the Carolliinae and Stenodermatinae subfamilies [20,24,25].

The genus *Artibeus* Leach, 1821 (Phyllostomidae: Stenodermatinae) comprises 13 species exhibiting a broad distribution throughout the Neotropics, while *Carollia* Gray, 1838 (Phyllostomidae: Carolliinae) includes 8 species, being the most prevalent in the Neotropical region [11,26]. In the cytogenetic context, both genera have several intriguing features, making this group an exciting model for evolutionary studies, including karyotype evolution. The great fruit-eating bat *Artibeus lituratus* exhibits 2*n* = 30♀/31♂ chromosomes, whereas Seba’s short-tailed bat *C. perspicillata* has 2*n* = 20♀/21♂, due to the presence of a multiple XY_1_Y_2_ male heterogametic system [24,27,28]. Intriguingly, other sex chromosome systems observed in Sternodermatinae evolved from an XY_1_Y_2_ system as observed in *Sturnira* with a compound system or Neo-XY related to *Artibeus* [29,30]. In *Artibeus,* the translocation event involved the X chromosome and a small acrocentric autosome [31], whereas in *C. perspicillata*, it involved a large autosome containing the 45S rDNA locus [29,32]. In situ hybridization experiments in *C. perspicillata* also demonstrate that eight chromosome pairs exhibit interstitial telomeric sites (ITS), representing the highest number of ITS yet documented for the family [32].

In this study, we selected two Phyllostomidae species, *Artibeus lituratus* and *Carollia perspicillata*, as models to isolate and characterize their satellitomes (i.e., the whole set of satDNA families). Firstly, to test the repetitive DNA content and genome size correlation in bats, we estimate the total content of satDNAs in the genome. Then we performed a comprehensive in situ hybridization analysis to determine the evolutionary trajectory of these satDNA sequences, and also microsatellite repeats, in those two species and also in phylogenetically related species. We select other Sternodermatinae species, including: the Gervais’s fruit-eating bat *Dermanura cinerea* (2*n* = 30 with a neo-XY system), the little-yellow-shouldered bat *Sturnira lilium* (2*n* = 30 with a neo-XY system), the tent-making bat *Uroderma bilobatum* (2*n* = 42 with a neo-XY system), and the brown tent-making bat *Uroderma magnirostrum* (2*n* = 36 with a neo-XY system), for this purpose. Comparative genomic hybridization (CGH) was performed to identify potential sex-specific regions in the multiple sex chromosomes of *C. perspicillata*.

## 2. Materials and Methods

### 2.1. Samples and Chromosomal Preparation

**Figure 1:**
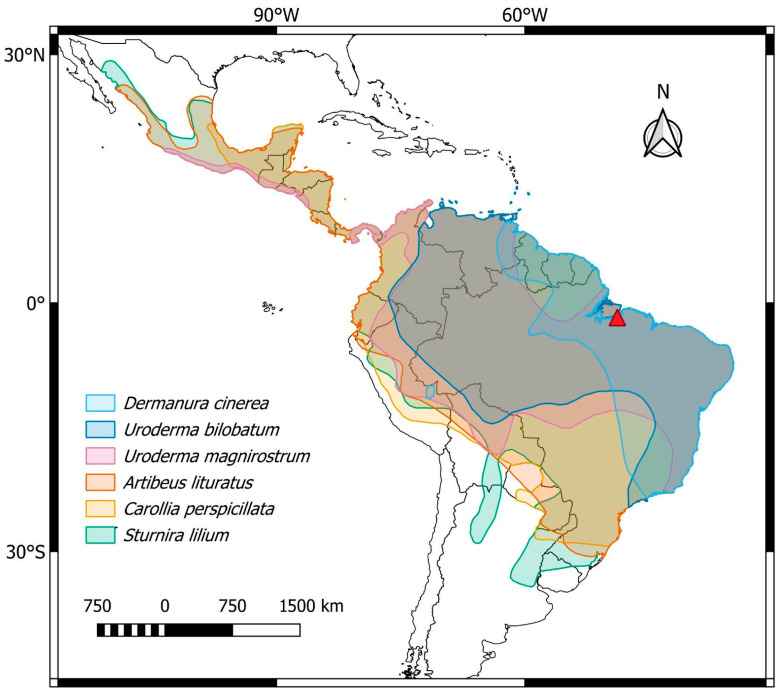
Central and South America, highlighting the general distribution of the phyllostomid bat species analyzed in the present work: *Dermanura cinerea* (light blue), *Uroderma bilobatum* (blue), *Uroderma magnirostrum* (pink), *Artibeus lituratus* (orange), *Carollia perspicillata* (yellow), and *Sturnira lilium* (green). The collection site is represented by the red triangle. Map created using QGIS 3.40.7 (http://qgis.org accessed on 12 June 2025).

Phyllostomid bats were collected in the municipality of Abaetetuba, Pará state, Brazil (1°46′03.2″ S 48°50′15.6″ W) using mist nets. The collection sites are shown in Table 1 and Figure 1. The mitotic cells were obtained from bone marrow preparations after colchicine treatment [33], and also from fibroblast cell cultures established from skin biopsies [34]. The specimens were collected with authorization from the National System for the Management of Genetic Heritage and Associated Traditional Knowledge (SISGEN-A96FF09) and the System of Authorization and Information about Biodiversity (SISBIO) under license number 92665-1. The experiments were conducted in accordance with the Ethics Committee on Animal Experimentation of the Universidade Federal de São Carlos, Brazil (Process number 7994170423). After sample collections, animals were fixed in 10% formalin, preserved in 70% ethanol, and deposited in the Zoology Collection of the Instituto Federal do Pará, Abaetetuba campus.

### 2.2. DNA Extraction and Genome Sequencing

Muscle samples of male individuals from *A. lituratus* and male and female individuals from *C. perspicillata* were used to obtain the genomic DNAs (gDNAs). The extraction process followed phenol-chloroform protocol [35], and the samples of one individual male of each species were then sequenced on the BGISEQ-500 platform (BGI Shenzhen Corporation, Shenzhen, China) (paired-end 2 × 150 bp), yielding 3 Gb of data for each species, representing ~1× coverage usually required for satellite assembly [36,37]. Raw reads were deposited on SRA-NCBI and are available under the accession numbers SRR32523217 for *A. lituratus* and SRR32523216 for *C. perspicillata*.

### 2.3. Bioinformatic Analyses and Satellitome Characterization

We isolated the satDNAs from raw reads of *A. lituratus* and *C. perspicillata* with the RepeatExplorer2 (RE2) pipeline [38]. To process the raw reads library, we used Trimmomatic (v. 033) [39] to remove low-quality reads and to crop the reads to be 100 base pairs long. After that, we randomly selected 2 × 500,000 reads from the trimmed libraries to prepare the library using the pipeline described by [36] and ran RE2 on the Galaxy server (v. 2.3.12.1) (https://repeatexplorer-elixir.cerit-sc.cz/galaxy). After the first iteration, and in each one thereafter, we filtered the previously inputted library to remove the putative satDNAs found using DeconSeq (v. 0.4.3.) [40] until no more satDNAs were found in the RE2 runs.

The catalogs of *A. lituratus* and *C. perspicillata*, (designated AliSatDNAs and CpeSatDNAs, respectively) were examined for additional tandemly repeated elements, including multigene families, utilizing the annotation function in Geneious (v. 7.3) [41], which yielded no further elements. Similarities between inter- and intra-species satellite DNAs were investigated using rm_homology_v2.py [42] and the “de novo assembly” function in Geneious 7.3 and classified according to [36], considering the percentage of similarity: around 95% the same satellite, while between 50 and 80% belonging to the same superfamily (SF).

We employed RepeatMasker (v. 4.1.9) [42] to assess the abundance and divergence of the two satellitomes, utilizing a random selection of 5,000,000 trimmed reads from each library with the “cross-match” tool. The satDNAs were arranged in descending order of abundance and renamed according to the protocol established by [36], utilizing the first letter of the genus, two letters from the specific epithet, and the size of the satDNA. The relative abundance of each satellite DNA was assessed by aligning selected reads with the complete catalog of satDNAs, where the ratio of mapped reads to the total number of nucleotides represented the relative abundance of each satDNA family. Divergence values were concurrently expressed as weighted average Kimura-2-parameter values for each sequence from the script calcDivergenceFromAlign.pl of the RepeatMasker [42]. The satDNA sequences of *Artibeus lituratus* were deposited in GenBank with accession numbers PV067726 to PV067729, while those for *Carollia perspicillata* were assigned numbers PV067174 to PV067183.

### 2.4. Primer Design and Conditions for Satellite Amplification

Primers were designed for five of the ten CpeSatDNAs and three of the four AliSatDNAs and then amplified by Polymerase Chain Reaction (PCR) (Table S1). The DNA template concentrations used were 0.1, 1, 10, and 100 ng. The amplification reaction consisted of the following cycles: 95 °C for 5 min for initial denaturation, followed by 34 cycles, each one consisting of denaturation at 95 °C for 45 s, primer annealing at 52–62 °C for 1 min, and extension at 72 °C for 1 min, with final extension at 72 °C for 5 min. The satDNA amplifications were checked in an electrophoretic agarose gel with 1 or 2% concentration and measured using a ThermoFisher NanoDrop spectrophotometer (ThermoFisher Scientific, Waltham, MA, USA). The remaining satDNAs with repeat unit lengths (RULs) smaller than 31 bp (CpeSat01-30, CpeSat02-24, CpeSat06-24, CpeSat08-21, CpeSat10-13, and AliSat01-19) were directly labeled with Cy3 at the 5′ end during the synthesis by ThermoFisher (ThermoFisher Scientific).

### 2.5. Probe Preparation and Fluorescence in Situ Hybridization (FISH)

The PCR-amplified satDNAs were labeled with Atto-550-dUTP using a nick translation protocol from Jena Bioscience (Jena, Germany). In addition, the microsatellites (A)n, (C)n, (CA)n, (GA)n, (GC)n, (TA)n, (CAA)n, (GAA)n, (TAA)n, (CGG)n, (CAC)n, (CAG)n, (CAT)n, (GAC)n, (GAG)n, and (TAC)n were also used as probes. These motifs were directly labeled with Cy3 at the 5′ end during synthesis (VBC Biotech, Vienna, Austria). Without the DNA template, telomeric sequences (TTAGGG)n were produced by PCR using the primers (TTAGGG)_5_ and (CCCTAA)_5_ according to [43]. These probes were labeled with Atto-550-dUTP using a nick translation protocol from Jena Bioscience (Jena, Germany). Following the guidelines provided by [44], high-stringency conditions were used for the fluorescence in situ hybridization (FISH) procedures. The 4,6-diamidino-2-phenylindole (DAPI) solution was used to counterstain all metaphase plates. Sequential G and C-banding was performed to identify the homologous and the sex chromosomes, according to [45,46], respectively.

### 2.6. Intraspecific Comparative Genomic Hybridization

Male and female gDNA of *C. perspicillata* were labeled using a nick translation protocol from Jena Bioscience (Jena, Germany) with Atto-550-dUTP and Atto-488-dUTP, respectively. We employed Cot-1 DNA isolated from the female gDNA of *C. perspicillata* to block common genomic repetitive regions. The final probe mix consisted of 10 μg of unlabeled female Cot-1 DNA and 500 ng of each male- and female-labeled gDNA, which was ethanol-precipitated, air-dried, and resuspended in 20 μL of hybridization buffer, pH 7.0. The comparative genomic hybridization (CGH) procedures were conducted using the protocol outlined in [47].

### 2.7. Microscopy and Image Analysis

A minimum of 30 metaphase spreads per individual were examined to validate the 2*n* and FISH findings. Images were acquired using an Axioplan II fluorescence microscope (Carl Zeiss, Jena GmbH, Jena, Germany) and processed using the ISIS software, version 5.5.9 (MetaSystems, Silver Spring, MD, USA).

## 3. Results

### 3.1. Satellitome Composition of A. lituratus and C. perspicillata

Using the RE2 pipeline, we identified 4 and 14 putative satDNAs for *A. lituratus* and *C. perspicillata*, respectively. From the 14 satDNAs in *C. perspicillata*, we revealed four putative satDNAs that exhibited around 95% similarity, which were considered the same satDNA. Considering this, the final catalog of *C. perspicillata* was composed of 10 CpeSatDNAs, with the repeat unit lengths ranging from 13 to 1681 base pairs and the A+T content varying between 33 and 60%, with an average of 50%. Among these ten, eight satDNA families showed more than 50% A+T content. The homology search by similarities between satellite DNAs found two superfamilies: superfamily 1 (CpeSat03-838 and CpeSat04-535) and superfamily 2 (CpeSat01-30, CpeSat02-24, CpeSat05-72, CpeSat07-1531, and CpeSat08-21) (Table 2). In *A. lituratus*, the satellite DNA sequences were all distinct, yielding four separate AliSatDNA families, with their repeat unit lengths ranging from 19 to 1388 base pairs, with the A+T content varying between 19.6% and 59.3% (Table 2). The repeat landscapes generated for male genomes of both species are shown in Figure S1. No significant similarity was found among the isolated satDNAs obtained from *A. lituratus* and *C. perspicillata* using the Basic local alignment search tool (BLAST)n at NCBI (https://blast.ncbi.nlm.nih.gov/Blast.cgi accessed on 7 July 2025).

### 3.2. Chromosomal Distribution of AliSatDNAs and CpeSatDNAs in A. lituratus and Carollia perspicillata

**Figure 2:**
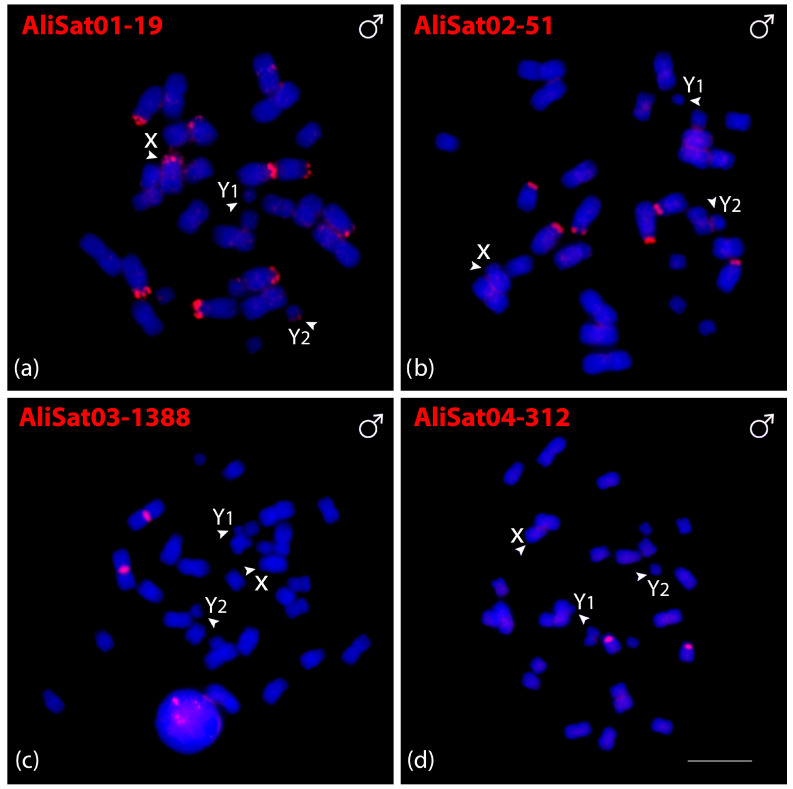
Male metaphasic chromosomes of *A. lituratus* showing hybridization signals of AliSatDNAs as probes (**a**–**d**). The name of each satellite DNA family is detailed in red in the upper left corner (Atto-550-dUTP labeled). The arrowheads indicate the sex chromosomes X, Y_1_, and Y_2_. Scale bar: 10 µm.

Fluorescence in situ hybridization (FISH) was employed to determine the chromosomal localization of AliSatDNAs and CpeSatDNAs. All four AliSatDNAs showed positive signals in some chromosomes of *A. lituratus* males (Figure 2a–d). AliSat01-19, AliSat02-51, and AliSat04-312 all exhibited hybridization signals concentrated in the terminal region. The AliSat01-19 was present in ten chromosomes, including four pairs of autosomes (5, 6, 7 and 9), and along with the X and Y_2_ chromosomes, AliSat02-51 was present on three pairs of autosomes (5, 6 and 7), and AliSat04-312 was dispersed on one chromosome pair (11). Meanwhile, AliSat03-1388 exhibited positive signals on the pericentromeric region of the largest metacentric chromosome pair (1) (Figure 2c).

**Figure 3:**
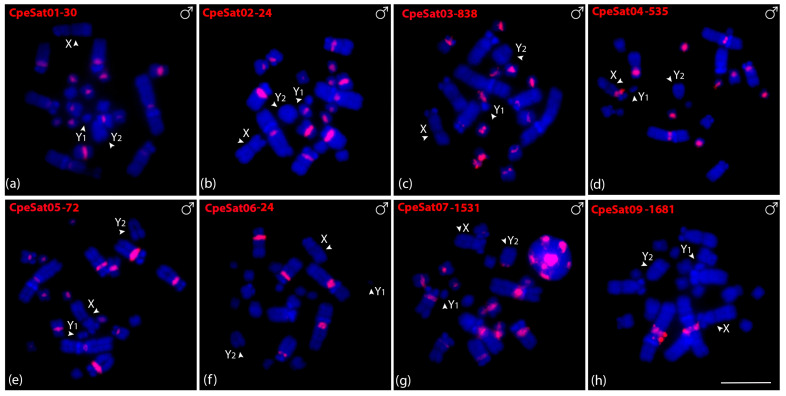
Male metaphasic chromosomes of *C. perspicillata* showing hybridization signals of CpeSatDNAs as probes (**a**–**h**). The name of each satellite DNA family is detailed in red in the upper left corner (Atto-550-dUTP labeled). The arrowheads indicate the sex chromosomes X, Y_1_, and Y_2_. Scale bar: 10 µm.

Eight out of ten CpeSatDNAs showed positive hybridization signals on *C. perspicillata* metaphase chromosomes in both males (Figure 3) and females (Figure S2). CpeSat01-30, CpeSat02-24, CpeSat03-838, CpeSat04-535, and CpeSat05-72 showed clustering mainly on the pericentromeric region of all chromosomes, except for some autosome pairs and the sex chromosomes (Figure 3a–e). Additionally, CpeSat04-535 and CpeSat07-1531 were clustered on the centromeric and pericentromeric regions of the X and Y_2_ chromosomes, respectively (Figure 3d,g), while CpeSat03-838 produces signals on the centromeric region, mainly on the small chromosomes (Figure 3c). Oppositely, CpeSat06-24 shows signals only on chromosome pairs 1, 2, and 3, and CpeSat09-1681 only on the pericentromeric region of pair 1 (Figure 3f,h). All the satellite DNAs of both *A. lituratus* and *C. perspicillata* were distributed on heterochromatic regions (Figure S3).

Cross-species hybridizations with the four AliSatDNAs in *C. perspicillata* chromosomes did not show any positive signals. Hybridizations using the ten CpeSatDNAs in *A. lituratus* chromosomes revealed positive signals for only two of them (CpeSat02-24 and CpeSat05-72) distributed mainly on the terminal region of all chromosomes (Figure S4a,b).

### 3.3. Chromosomal Distribution of Microsatellite and Telomeric Repeats in A. lituratus and C. perspicillata

**Figure 4:**
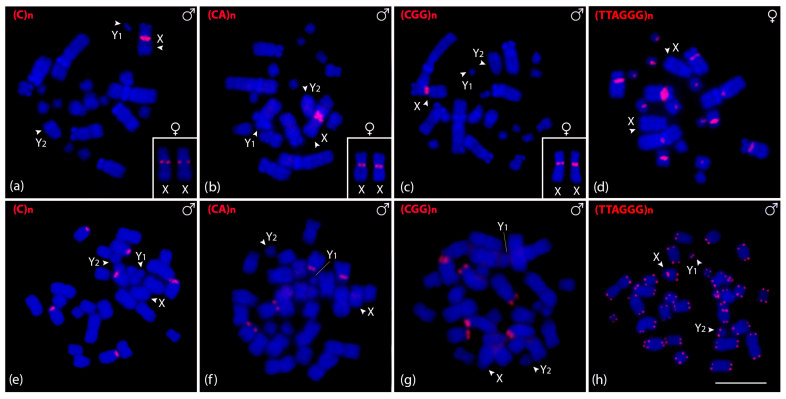
Male and female metaphasic chromosomes of *C. perspicillata* (**a**–**d**) and male of *A. lituratus* (**e**–**h**) showing hybridization signals of CpeSatDNAs as probes. The name of the repeat motif is detailed in the upper left corner. The arrowheads indicate the sex chromosomes X, Y_1_, and Y_2_. Scale bar: 10 µm.

We also in situ mapped microsatellite and telomeric motifs to determine their chromosomal locations. Only three of the sixteen microsatellite repeat motifs showed positive signals on *C. perspicillata* chromosomes. (CA)n, (C)n, and (CGG)n motifs showed hybridization signals in female metaphase chromosomes and one signal in males, corresponding to the X chromosome (Figure 4a–c). Intriguingly, the telomeric probe (TTAGGG)_5_ hybridized onto the pericentromeric region instead of the telomeric regions, except for the sex chromosomes (Figure 4d). We then tested the three microsatellite motifs that showed positive signals on *C. perspicillata* in *A. lituratus.* (CA)n and (CGG)n displayed the same hybridization pattern in the terminal region of six chromosomes, while (C)n were clustered in four chromosomes (Figure 4e–g). The telomeric probe (TTAGGG)_5_ showed hybridization signals on the terminal region of all chromosomes. Furthermore, we identified the interstitial telomeric signal (ITS) on chromosome pair 4 in *A. lituratus* (Figure 4h).

### 3.4. Cross-Species Mapping of satDNAs and Chromosomal Distribution of Microsatellites and Telomeric Repeats in Other Bat Species

All AliSatDNAs and CpeSatDNAs were in situ mapped in other Sternodermatinae species (*D. cinerea*, *S. lilium*, *U. bilobatum*, and *U. magnirostrum*) to check the presence of conserved satDNAs. Among all, only CpeSat02-24 and CpeSat05-72 presented positive signals (Figure S4). In *D. cinerea* and *S. lilium*, CpeSat02-24 and CpeSat05-72 showed clustering in the terminal region of all chromosomes, similar to those observed for *A. lituratus*. In *U. bilobatum*, besides CpeSat02-24, CpeSat05-72 presented strong signals on the X chromosome and on the terminal region of all chromosomes, and in *U. magnistrostum*, on the centromeric region of six autosome pairs (5, 6, 8, 10, 13 and 14), and on the X chromosome. Comparative hybridization using the general telomeric repeats (TTAGGG)n presented a pattern similar to that observed for the CpeSatDNAs (Figure S4c,f,i,l and Figure 4h).

**Figure 5:**
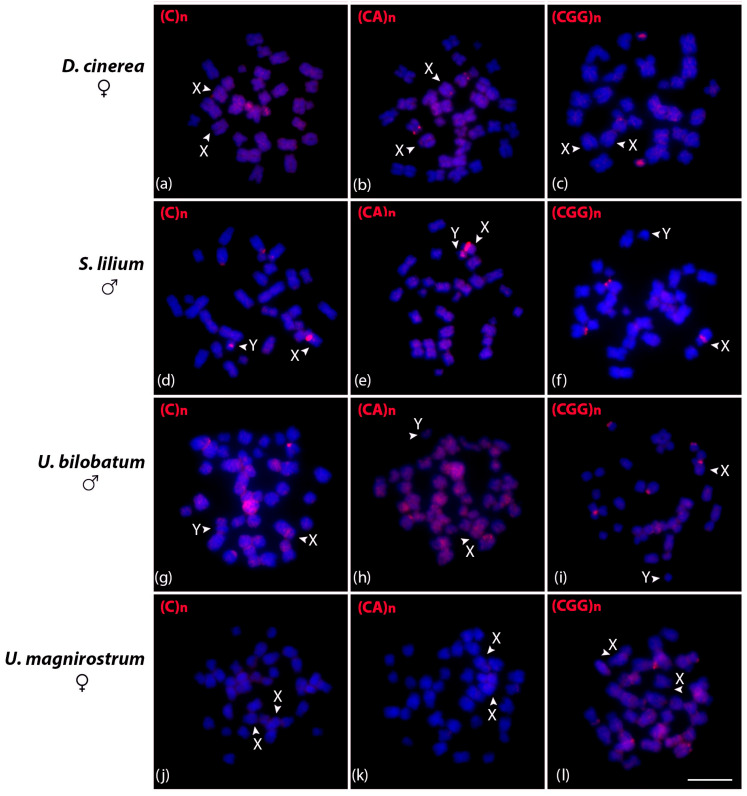
Metaphasic chromosomes of *D. cinerea* female (**a**–**c**), *S. lilium* male (**d**–**f**), *U. bilobatum* male (**g**–**i**), and *U. magnirostrum* female (**j**–**l**) after in situ hybridization experiments with (C)n, (CA)n, and (GCC)n microsatellite probes indicated in red in the upper left corner. Scale bar: 10 µm.

The three satDNA repeats identified in *C. perspicillata* were additionally investigated in *D. cinerea*, *S. lilium*, *Uroderma bilobatum*, and *U. magnirostrum* (Figure 5). In *D. cinerea* females, (C)n and (CGG)n were mapped in one small chromosome pair and (CA)n on the short arm interstitial region of a small submetacentric pair (Figure 5a–c). The (C)n and (CA)n in the *S. lilium* male were clustered in the pericentromeric region of both X and Y, and (CGG)n only on the X chromosome. Additionally, we observed a centromeric signal in a large acrocentric pair using the (C)n and (CGG)n probes (Figure 5d–f). In the *U. bilobatum*, (C)n and (CA)n were clustered in the centromeric region of one chromosome pair and (CGG)n on two pairs, and also on the X (Figure 5g–i). The (C)n and (CA)n repeats did not present positive signals in *U. magnirostrum* chromosomes, and (CGG)n clustered in the terminal region of the short arms of two chromosome pairs (Figure 5j–l).

### 3.5. Intraspecific Genomic Hybridization in C. perspicillata

**Figure 6:**
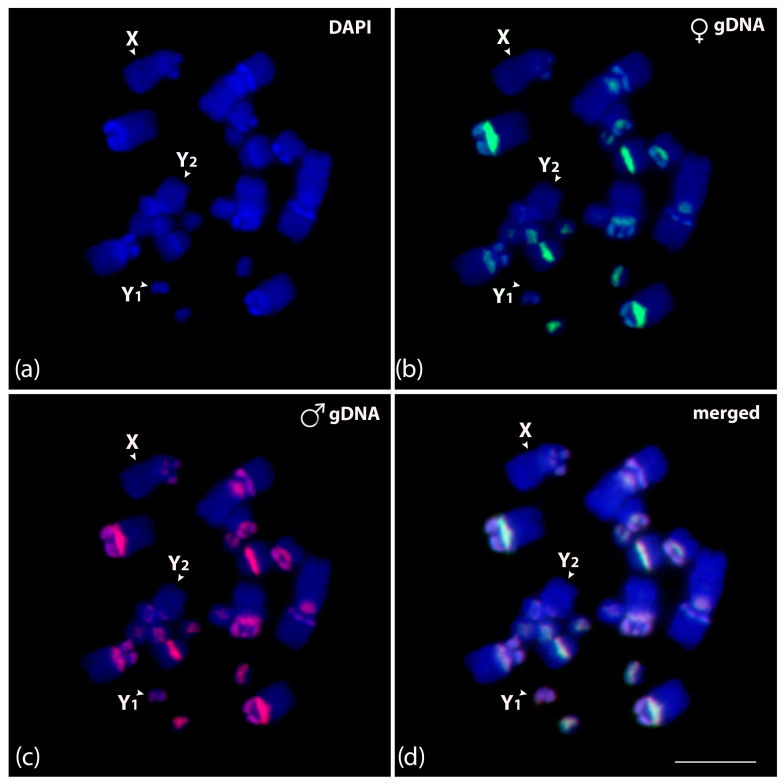
Mitotic chromosome spreads of *Carollia perspicillata* male (**a**–**d**) after intraspecific genomic hybridization with female- and male-derived genomic probes. (**a**) DAPI-stained chromosomes (blue); (**b**) hybridization pattern for the female genomic DNA in green (Atto-488-dUTP labeled); (**c**) hybridization pattern for the male genomic DNA in red (Atto-550-dUTP labeled); and (**d**) merged images for DAPI-stained and both genomic DNA probes. According to morphology, the sex chromosomes are identified and indicated in each chromosome spread. Scale bar: 10 µm.

To identify putative sex-specific regions in multiple systems, we compared male and female genomes of *C. perspicillata*. The results of genomic comparison detected overlapped centromeric and pericentromeric regions on all chromosomes, except for X, Y_1_, and Y_2_. An exclusive accumulation of male repeats was observed on the Y_1_ chromosome (Figure 6).

## 4. Discussion

In this study, we examined the repetitive DNA content of flying mammals at the genomic and chromosomal levels. The in silico data allowed us to identify two libraries composed of a few satellite DNA sequences, representing a low proportion of this repetitive sequence class in *A. lituratus* and *C. perspicillata* genomes; some of which present a direct association with telomeric sequences. The in situ mapping demonstrated the distribution of satDNAs in the telomeric and pericentromeric regions and microsatellite repeats in adjacent regions to the 45S rDNA cluster. The findings presented here revealed new occurrences between telomeric sequences and satellite DNA association in mammals, thereby providing insight into the involvement of these repeats in the evolutionary dynamics within these species.

### 4.1. General Features of A. lituratus and C. perspicillata Satellitomes

Prior characterization of satDNA in mammals revealed satellitomes consisting of a limited number of families, accounting for a minor fraction of repetitive DNA content (about 1% of the genome) [48,49]. The exception was related to rodent species of the genus *Microtus*, in which 17 satDNA families were identified, corresponding to 6% of the genome [50]. In bats, a significant presence of satDNAs was also observed in *Perimyotis subflavus* (Vespertilionidae), accounting for 6% of its genome [51]. In other bat species, however, the presence of some TE classes is higher in the genomic representation, like in *A. lituratus* (Phyllostomidae), in which about 15% of the genome is represented by the long interspersed element-1 (LINE-1) [51]. In our context, satDNAs constituted minor fractions of the genome, accounting for 0.014% in *A. lituratus* and 0.073% in *C. perspicillata* (Table 2), reinforcing that bats possess even smaller amounts of this repetitive DNA class than other mammals and how the satDNA content can vary within this group.

**Figure 7:**
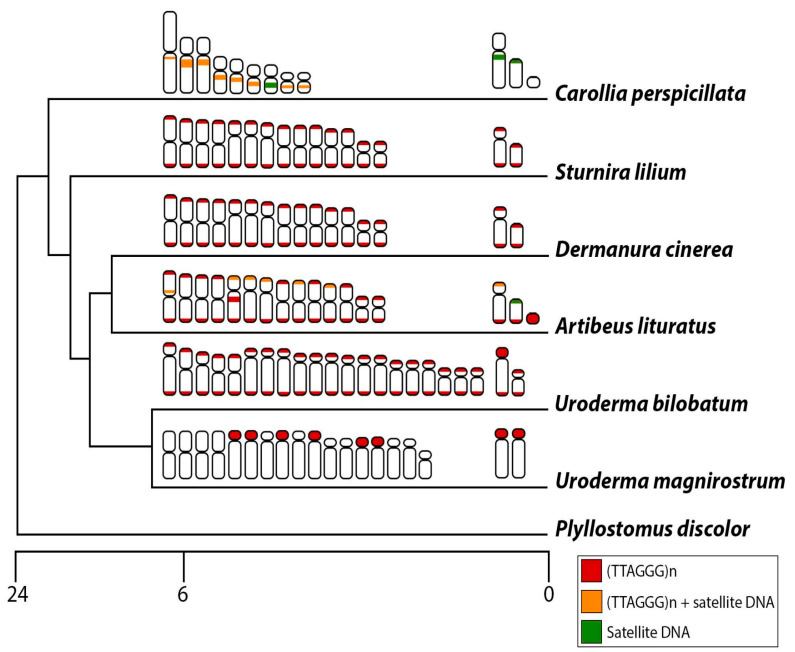
Schematic representation of the phylogenetic relationship of phyllostomid species analyzed in the present work and the distribution of telomeric repeats and satellite DNA: exclusive distribution of (TTAGGG)_5_ (red), (TTAGGG)_5_ and satellite DNA (orange), and exclusive distribution of satellite DNA (green). The scale bar corresponds to million years ago (MYA).

In most instances, satDNAs may be conserved among closely related organisms, including species within the same genus [50,52] or even across the same family or subfamily [50,53,54,55], as posited by the library hypothesis [56]. However, in some reports, arrays of satDNAs presented in the heterochromatic regions were considered a chromosome- or species-specific variant [57,58]. Here, we observed that the whole library was species-specific since the in-silico approach could not identify any common satellites between *A. lituratus* and *C. perspicillata*. These findings probably can be associated with the estimated divergence time of around 21.1 million years ago between these species (median time calculated by [59]) (Figure 7), along with the concept that satDNAs have been considered one of the most rapidly shifting repetitive DNAs in the genome [5]. Since we only analyzed one species per genus, comparative studies centered on satDNA analysis in other bat species within the *Artibeus* and *Carollia* genera could shed light on the conservation of these repeats.

Satellitome studies also have demonstrated that centromeric-associated repeats exhibit rapid changes and significant variability across closely related species, with mutation fixation facilitating the emergence of species-specific centromeric sequences [60,61,62]. Studies in mammals have connected satellite sequences to centromeric regions [48,53,63]. Possible CENP-B box-like motifs were found in the Neotropical deer *Mazama gouazoubira* and the Florida manatee subspecies *Trichechus manatus latirostris* [53,63]. The mostly satellite DNAs of *C. perspicillata* were found in the (peri)centromeric region of several chromosome pairs; however, BLAST analyses did not find any of them to be associated with putative centromeric satellite DNA or with any other repetitive sequence already described. Besides satellite DNAs, centromere-associated elements in plants also include some retrotransposons (reviewed in [64]). In this sense, investigations focused on the accurate centromere motif identification methods or even the identification of other repeats as main components of bat centromeres (e.g., LINE-1 retrotransposons, since it was largely found in the centromeric region [65]) can better elucidate the centromeric organization in bats.

### 4.2. Heterochromatin, Telomeric Repeats, and Satellite DNA Association

From plants to animals, the centromeres are frequently surrounded by pericentromeric regions enriched in constitutive heterochromatin (CH) and several different types of highly repetitive DNAs [66]. Among bats, incredible amounts of heterochromatin in interstitial chromosomal regions were reported [25,67,68], and the in situ mapping in phyllostomid bats demonstrates the presence of LINE elements [65] and satellite repeats, including telomeric sequences associated with these regions (present study).

According to our data, the distribution of the majority of CpeSatDNAs also coincides with the pericentromeric C-heterochromatin bands and telomeric motifs in *C. perspicillata*. In fact, CpeSat02-24 consists of the canonical telomeric repeat (TTAGGG)n (confirmed by in silico and in situ approaches). These repeats are also part of the CpeSat05-72 and homologies between both classified CpeSat02-24 and CpeSat05-72 as the same superfamily. The association of these satDNAs and telomeric sequences is also supported by fluorescence in situ experiments with CpeSat05-72, presenting signals associated with telomeric patterns in different bat species. Even in *U. magnirostrum*, with prominent centromeric signals in several chromosome pairs of CpeSat05-72, were also represented by telomeric repeats (Figure S4).

Telomeres in bats have been considered intriguing, especially regarding their unusual distribution across the chromosomes, frequently found in pericentromeric regions associated with CH [32]. In *C. perspicillata*, the chromosomal termini lack telomeric signals but exhibit significant accumulation in the pericentromeric region [32,69] (present work). This pattern was also identified in *U. magnistrum*, where telomeric repeats are highly amplified in the pericentromeric region of some chromosome pairs. Ref. [32], analyzing the distribution pattern of telomeric signals in phyllostomid bats, identified basal karyotypes possessing mainly telomeric signals. In fact, *C. perspicillata* and species of the *Uroderma genus* present the most divergent karyotypes within Phyllostomidae (reviewed in [20]). In the present study, terminal hybridization signals were observed in *A. lituratus*, *D. cinerea*, *S. lilium*, and *U. bilobatum* (Figure S4 and Figure 7). Even though *U. bilobatum* presents telomeric signals and *U. magnirostrum* centromeric accumulation of telomeric repeats, *U. bilobatum* species exhibit a more evolutionary derived karyotype compared to *U. magnirostrum* [68]. Considering the recent divergence (around 5 million years ago between *U. bilobatum* and *U. magnirostrum*), the telomeric repeats present very different distributions between *Uroderma* species, reinforcing how particular the evolution of telomeric sequence is in bats.

Regarding the absence of terminal signals using the canonical telomeric sequence, we must consider several possibilities, including the prospect of different sequences performing the role of chromosome stability, as seen in some insects [70,71], or even a different organization, similar to what has been reported in marsupials [72]. Furthermore, it is possible that the number of telomere repeats is below the detection limit for the method used, i.e., FISH. The observation in human molecular cytogenetics that even centromeric repeats present on a derived chromosome (detected by microdissection and reverse FISH) may not be detectable by FISH could support this possibility [73].

The telomeric repeats and satellite DNA association have been reported for some fish and plant species. In the Sparidae species *Pagellus erythrinus* (Acanthuriformes: Sparidae), sequencing analyses of Dra I satellite DNA indicated an association between satDNA and telomeric sequences, and in situ experiments revealed a subtelomeric distribution [74]. Similarly, a telomere-associated satellite DNA (RUSI) isolated from the Iberian sorrel *Rumex induratus* (Polygonales: Polygonaceae) contains different variants of degenerate telomere motifs, and in situ experiments revealed that the RUSI repeats are not only located at the subtelomeric, but also the centromeric region of some chromosomes [75]. On the other hand, in Thomas’s pine vole, *Microtus thomasi* (Rodentia: Cricetidae), telomeric sequences were massively amplified in the pericentromeric heterochromatin [76,77] and additionally, two variants of the canonical telomeric repeat (MthoSat4-6) were found [50]. Here we report two sequences, one corresponding to CpeSat02-24 and the other to CpeSat05-72, classified as the same superfamily (high similarity, but with some differences in the sequence content). It is reasonable that once these sequences are no longer located in the telomeric region, fulfilling their function of chromosome stability and protection, they tend to degenerate. Thus, mutational events may be the main cause for the presence of these variations of satDNA sequences.

In addition to telomeric studies, bats are also well characterized regarding the in situ mapping of multigenic rDNA families. While most bat species typically possess one autosomal pair containing the 45S rDNA cluster, episodes of sex-autosome translocation may result in this cluster being located on the sex chromosomes, similar to observations in *Carollia*, or distributed across multiple chromosomal sites, as noted in *Artibeus* [32]. The microsatellite repeats (CA)n, (C)n, and (CGG)n showed positive signals associated with the 45 rDNA cluster in *C. perspicillata*. This pattern was also observed in *A. lituratus* for (C)n and (CGG)n repeats. The association between multigene families and simple sequence repeats (SSRs) is very common and has been reported in other groups. In the armored catfish *Rineloricaria latirostris* (Siluriformes: Loricariidae) and in the freshwater stingray *Potamotrygon schroederi* (Myliobatiformes: Potamotrygonidae), SSRs were detected associated with the 5S and 45S rDNA genes, respectively [78,79]. Here, besides the microsatellites, the AliSat01-19 and AliSat02-51 satellites presented positive signals coincident with the location of the 45S rDNA sites in *A. lituratus*, reinforcing the view that the secondary constriction regions of bats are enriched in microsatellite and satellite DNAs. Variation in the size and composition of the intergenic spacer of rDNA is frequently associated with the presence of several repetitive DNA classes [80]. In this sense, detailed investigations, including the characterization of these regions in bats, can confirm the results obtained from in situ mapping.

### 4.3. Repetitive DNAs and the Evolution of Sex Chromosomes in Bats

Most Carolliinae and Sternodermatinae species are characterized by having multiple or compound sex chromosome systems with a high frequency of chromosomal rearrangements [27,29,81,82,83,84]. In multiple sex chromosomes, where a Y fission triggers two chromosomes, named Y_1_ and Y_2_ chromosomes, the true male sex chromosome of the system is Y_1_, while Y_2_ represents the autosome involved in the translocation/fission. In accordance with earlier cytogenetics reports that the Y_1_ is the most likely carrier of the male-specific genes [29,85], the results of the intraspecific CGH experiment in *C. perspicillata* highlight a male-specific region on the Y_1_ chromosome. *A. lituratus* harbors the same system as *C. perspicillata*; however, with a different origin, since different chromosomes are involved in the origin [81]. We hypothesized a similar pattern with the Y_1_-carrying male-specific sequences; however, we couldn’t assume this pattern, since no female gDNA was available for CGH experiments. Intraspecific CGH in XY systems also reveals male-specific regions in rodents and primates [47,86], marking this as the first intraspecific hybridization in a multiple sex chromosome system in mammals.

The analysis of chromosomal distribution of repetitive DNAs is sparse and limited in bats [65], especially with a focus on the sex chromosomes. In the present work, satellite distribution of repetitive DNAs in multiple sex chromosomes includes AliSat01-19 in *A. lituratus*, clustered in some autosome pairs but also on the X and Y_2_ chromosomes, being absent on the Y_1_. In this case, the enrichment of satellite DNA on both X and Y_2_ chromosomes can be related to the two autosomal segments involved in the translocation, reinforcing their common origin. In *C. perspicillata*, only CpeSat04-535 and CpeSat07-1531 were clustered in the X and Y_2_, respectively. So, specifically for CpeSat04-535, the loss of repetitive sequences after the fission event appears to be a characteristic of this multiple chromosome system, since few sequences were accumulated on the X chromosome and were absent or with few copy numbers on the Y chromosomes. Besides satDNAs, microsatellite repeats are the only types of sequences investigated in this study that are grouped on the X chromosomes of *C. perspicillata*, related to the rDNA 45S cluster as mentioned earlier. In this case, it is feasible that this accumulation of repetitive sequences may stabilize the chromosomal rearrangement, since they were mapped at the fusion point between the original X chromosome and the autosome (Figure 4a–c).

Compound sex chromosome systems in bats are characterized by a secondary translocation that reorganized the sex chromosomes through centric fusion, originating neo-sex chromosome systems. Between *Artibeus planirostris* and *Sturnira lilium*, the only difference observed is related to the sex chromosome system involving a reciprocal X/autosome translocation, characterizing a neo-XY system [30]. Here, in *S. lilium*, we observed a significant accumulation of microsatellite repeats on the sex chromosomes. While (C)n was distributed in both autosomal and sex chromosomes, (CA)n appears to be a sex-chromosome-specific marker present in both X and Y. On the other hand, the pattern present with (CGG)n indicates a loss of this sequence or diminution in copy number on the Y. In grasshoppers, other repetitive DNAs analyzed on neo-sex chromosomes highlight an intensive accumulation reinforcing the differentiation and high turnover events of neo-sex chromosomes [87,88,89,90]. Similar to those found in insects, bats also represent a group with high turnovers of neo-sex chromosomes. In this sense, studies with a focus on the characterization of other repetitive sequences can shed light on the molecular differentiation of the few explored systems in this mammal group.

Although *Uroderma* species do not exhibit enrichment of microsatellite repeats, similar to those repeats found in *S. lilium*, telomeric sequences were highly prevalent on the X chromosomes of *U. bilobatum* (Figure S4). In *U. magnirostrum*, the large accumulation of telomeric sequences in the centromeric region of several autosome pairs is also present on the X chromosome. Since we have not performed in situ hybridization on male chromosomes, it is not possible to confirm whether its accumulation was lost in the Y; however, an expected pattern similar to that of *U. bilobatum* is possible. Taken together, our study points to a significant contribution of different repetitive DNAs in the sex chromosome differentiation in bats. Moreover, investigations involving other microsatellite repeats and the characterization of satDNA libraries from other bat species might enhance the understanding of karyotypic evolution related to the processes that trigger chromosomal rearrangement events and sex chromosome differentiation in mammals.

## 5. Conclusions

This study characterized satellite DNA sequences and analyzed the distribution of repetitive repeats for the first time from multiple phyllostomid bat species, which have been poorly investigated. Our study confirmed the hypothesis that the reduced genome size in bats is possibly associated with a loss of repetitive sequences, especially satellite DNAs. Furthermore, our study also demonstrated the presence of a few satellite families that were associated with heterochromatic regions, similar to what has been reported previously for other mammals, thereby underscoring the significance of satellite DNA as a principal content of heterochromatin. We also demonstrated that in two of our species, only a few satellites accumulated on the sex chromosomes, and in *C. perspicillata*, some satellites were associated with telomeric sequences. The absence of terminal signals relative to canonical telomeric repeats in some bat species remains intriguing. Future investigations with whole-genome sequencing can offer previously unknown details about this organization.

## Figures and Tables

**Table 1:** Individuals of the Phyllostomidae family analyzed in the present work, including their respective diploid numbers (2*n*), sex chromosome systems (SCS), and number of sampled individuals (N). The asterisk (*) represents the target species for the satellitome analyses.

Subfamily	Species	2*n*	SCS	N
Sternodermatinae	*Artibeus lituratus* *	2*n* = 30♀/31♂	XY_1_Y_2_	2♂
	*Dermanura cinerea*	2*n* = 30♀♂	neo-XY	1♀
	*Sturnira lilium*	2*n* = 30♀♂	neo-XY	1♂
	*Uroderma bilobatum*	2*n* = 42♀♂	neo-XY	2♂
	*Uroderma magnirostrum*	2*n* = 36♀♂	neo-XY	1♀
Carolliinae	*Carollia perspicillata* *	2*n* = 20♀/21♂	XY_1_Y_2_	3♀/5♂

**Table 2:** General features of *Artibeus lituratus* (AliSatDNAs) and *Carollia perspicillata* (CpeSatDNAs) satellitomes, including the classification in superfamilies (SF).

SF	satDNA Family	Monomer Size	Abundance	Divergence	A+T (%)
	AliSat01-19	19	0.010222866	23.74	47.4
	AliSat02-51	51	0.003122652	11.58	19.6
	AliSat03-1388	1388	0.000778856	4.21	57.3
	AliSat04-312	312	0.000475007	4.21	59.3
2	CpeSat01-30	30	0.030872539	6.02	60
2	CpeSat02-24	24	0.019716908	15.31	50
1	CpeSat03-838	838	0.008007920	3.44	55
1	CpeSat04-535	535	0.006001807	5.38	56
2	CpeSat05-72	72	0.005138635	8.8	56
	CpeSat06-24	24	0.002154351	7.42	33
2	CpeSat07-1531	1531	0.000667462	15.53	58
2	CpeSat08-21	21	0.000527628	19.33	38
	CpeSat09-1681	1681	0.000375722	7.47	53
	CpeSat10-13	13	0.000085140	9.96	38

## Data Availability

The datasets generated during and/or analyzed during the current study are available from the corresponding author upon reasonable request. The datasets generated and analyzed during the current study are available in the GenBank repository under the accession numbers PV067726-PV067729 and PV067174-PV067183.

## Supplementary Materials

The following supporting information can be downloaded at: https://www.mdpi.com/article/10.3390/biom15091248/s1. Figure S1. Repeat landscape of (a) AliSatDNAs and (b) CpeSatDNAs in the male genome of *Artibeus lituratus* and *Carollia perspicillata* species, respectively. Figure S2. Female metaphasic chromosomes of *C. perspicillata* showing hybridization signals of CpeSatDNAs as probes (a–i). The name of each satellite DNA family is detailed in the upper left corner in red (Atto-550-dUTP labeled). The arrowheads indicate the sex chromosomes X, Y_1_, and Y_2_. Scale bar: 10 µm. Figure S3. Male and female metaphasic chromosomes of bat species analyzed in the present work after C-positive heterochromatin detection: (a) *Artibeus lituratus*, (b) *Carollia perspicillata*, (c) *Dermanura cinerea*, (d) *Sturnira lilium*, (e) *Uroderma bilobatum*, and (f) *Uroderma magnirostrum*. Scale bar: 10 µm. Figure S4. Male and female metaphasic chromosome plates of *A. lituratus*, *D. cinerea*, *S. lilium*, *U. bilobatum*, and *U. magnirostrum* for comparative hybridizations using the Telomeric (TTAGGG)n and CpeSatDNAs repeats. Scale bar: 10 µm. Table S1: Designed primers for all satellite DNAs analyzed in the present study. The satDNAs with repeat unit length smaller than 31 bp were directly labeled with Cy3 at the 5′ end during the synthesis (*).

## Abbreviations

The following abbreviations are used in this manuscript:

TE	Transposable elements
satDNA	Satellite DNA
rDNA	Ribosomal DNA
2n	Diploid number
ITS	Interstitial Telomeric Sites
gDNA	Genomic DNA
RE2	RepeatExplorer2
PCR	Polymerase Chain Reaction
RUL	Repeat Unit Length
FISH	Fluorescence in situ Hybridization
CGH	Comparative Genomic Hybridization
CH	Constitutive Heterochromatin
SSRs	Simple Sequence Repeats
