# Supplementary material for: Repetitive DNAs and Karyotype Evolution in Phyllostomid Bats (Chiroptera: Phyllostomidae)
Source: Biomolecules. 2025 Aug 29;15(9):1248. doi: 10.3390/biom15091248 (PMC12467311; doi:10.3390/biom15091248)
Supplement: Supplementary file 1 [file biomolecules-15-01248-s001.zip › biomolecules-3831883-supplementary.pdf]

Supplementary Materials

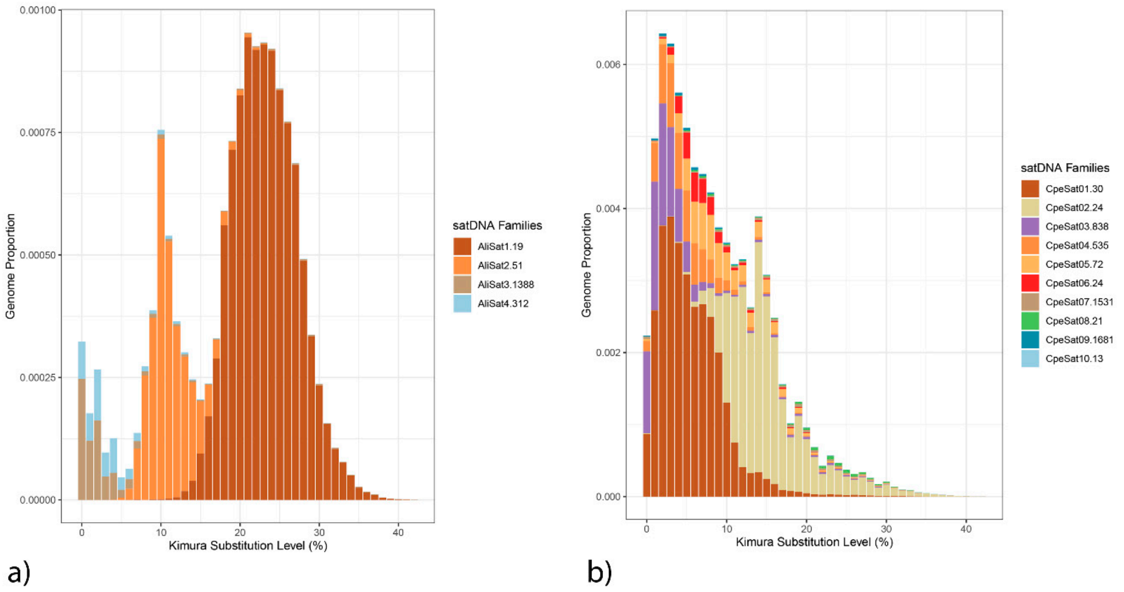

Figure S1. Repeat landscape of a) AliSatDNAs and b) CpeSatDNAs in the male genome of *Artibeus lituratus* and *Carollia perspicillata* species, respectively

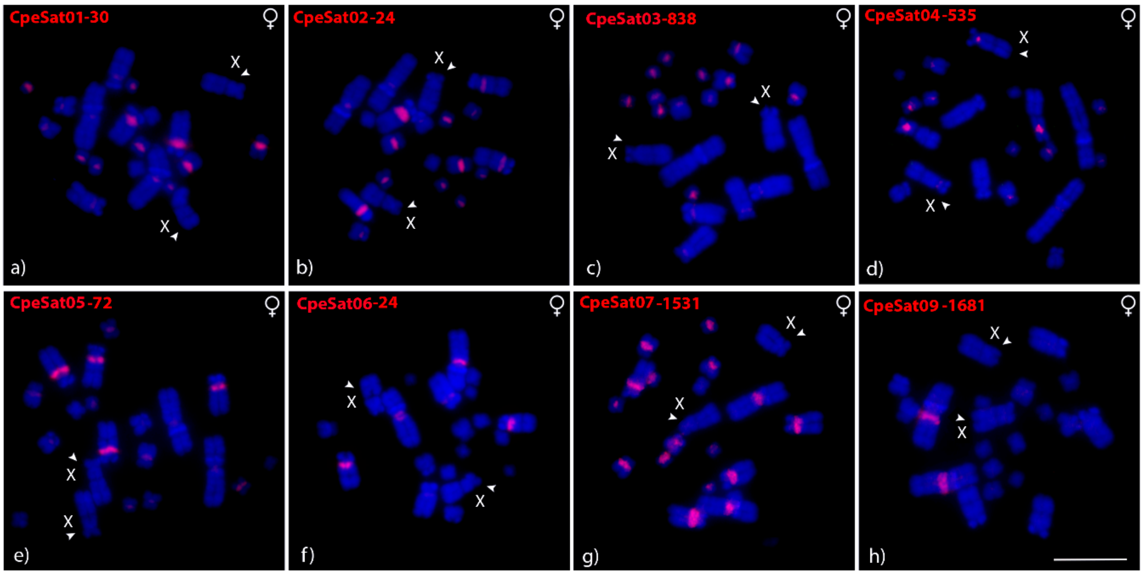

Figure S2. Female metaphasic chromosomes of *C. perspicillata* showing hybridization signals of CpeSatDNAs as probes (a-i). The name of each satellite DNA family is detailed in the upper left corner in red (Atto-550-dUTP labeled). The arrowheads indicate the sex chromosomes X, Y_1, and Y_2. Scale bar: 10 μm

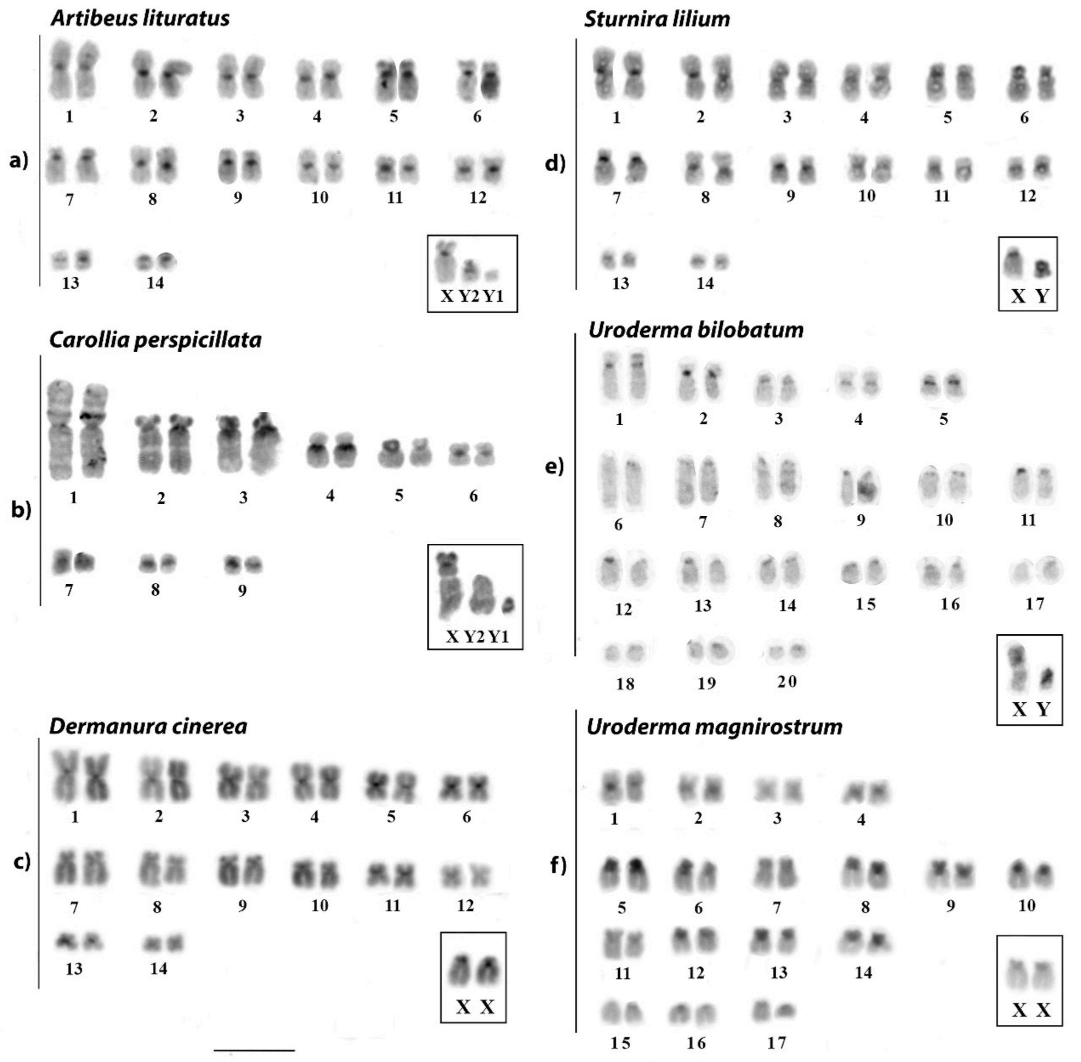

Figure S3. Male and female metaphasic chromosomes of bat species analyzed in the present work after C-positive heterochromatin detection: a) *Artibeus lituratus,* b) *Carollia perspicillata,* c) *Dermanura cinerea,* d) *Sturnira lilium,* e) *Uroderma bilobatum,* and f) *Uroderma magnirostrum*. Scale bar: 10 μm.

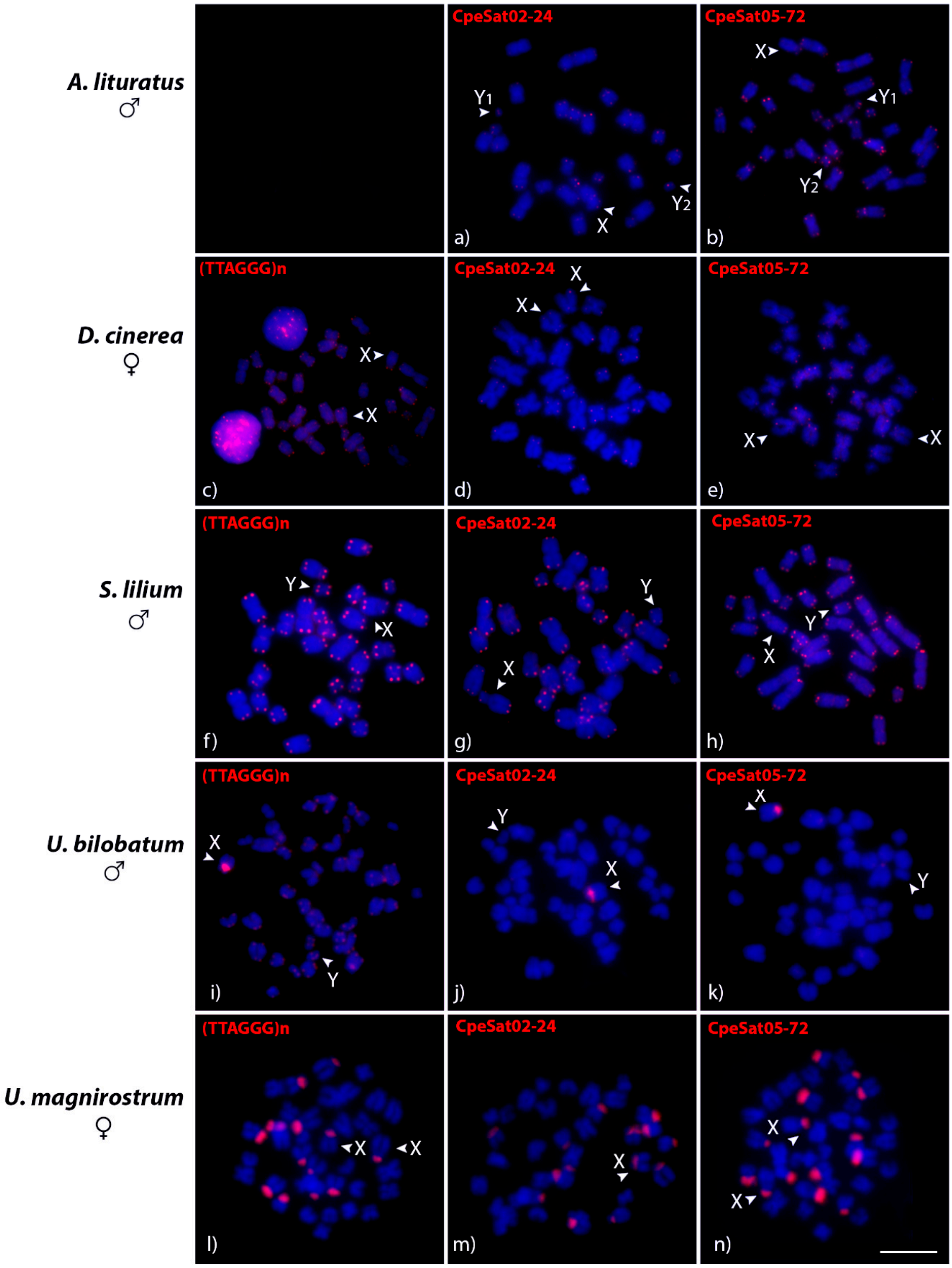

Figure S4. Male and female metaphasic chromosome plates of *A. lituratus*, *D. cinerea*, *S. lilium*, *U. bilobatum*, and *U. magnirostrum* for comparative hybridizations using the Telomeric (TTAGGG)n and CpeSatDNAs repeats. Scale bar: 10 µm.

Table S1. Designed primers for all satellite DNAs analyzed in the present study. The satDNAs with repeat unit lengths (RULs) smaller than 31 bp were directly labeled with Cy3 at the 5′ end during the synthesis (*).

Satellite DNA	Primer Forward	Primer Reverse
AliSat01-19*	AGAGATGACTGTGCAGGTG-Cy3	
AliSat02-51	GTTGTGGTGGAGCGGCCG	GCCGGCTGCTTGACCCGG
AliSat03-1388	CGGATAAACACTTTCCAACTGA	CTCCCTGGGTATCTCATCTG
AliSat04-312	TAAACAGTCCCCCAGGTTGC	CAGGGTTGATATTTTCCTCAG
CpeSat01-30*	TAGTGTTAGGGTTAGGGTTAC GATTACTGT-Cy3	
CpeSat02-24*	AACCCTAACCCTAACCCTAAC CCT-Cy3	
CpeSat03-838	CGTTCGCTCGGTAGCTGA	CTCCATGGACAAGGCACTC
CpeSat04-535	AGCACTTCTCGGCTAAGAA	CTTTGTTAAGACTGTGAGAG
CpeSat05-72	CTACGGTATTGTTTAGAGCT	CCTAACACTAAACGTAACC
CpeSat06-24*	ACCCGAACCCGAACCCGAACC CGA-Cy3	
CpeSat07-1531	CCTGGCGATAACCCTAGC	GGTTAGGTTTAGGGTACGAG
CpeSat08-21*	GGGCTAGGGCTAGGGCTGT TA-Cy3	
CpeSat09-1681	GGTGAAGTGGATCATAACG	CTTCACATTGACACTCTAG
CpeSat10-13*	TGCCCTCTCACTG-Cy3	
